# Positive Mental Health from the perspective of Iranian society: A qualitative study

**DOI:** 10.12688/f1000research.13394.2

**Published:** 2018-02-16

**Authors:** Arash Mirabzadeh, Monir Baradaran Eftekhari, Katayoun Falahat, Homeira Sajjadi, Meroe Vameghi, Gholamreza Ghaedamini Harouni

**Affiliations:** 1 Social Determinant of Health Research Center, University of Social Welfare and Rehabilitation Sciences, Iran, Tehran, 1985713834, Iran; 2 Department of Psychiatry, University of Social Welfare and Rehabilitation Sciences, Iran, Tehran, 1985713834, Iran; 3 Deputy for Research and Technology, Ministry of Health and Medical Education, Tehran, 1467664961, Iran; 4 Social Welfare Management Research Center, University of Social Welfare and Rehabilitation Sciences, Iran, Tehran, 1985713834, Iran

**Keywords:** Positive Mental Health, qualitative study, focus group discussion

## Abstract

**Background**: According to the World Health Organization, mental health relates, not only to the absence of mental disorder, but also to Positive Mental Health. Studies have shown that promoting positive mental health, not only reduces the prevalence and incidence of mental disorders, but also affects the process of treatment and reduces related burden. However, this concept has different interpretations in different cultures, and in many societies, mental health is still considered the absence of mental illness. Thus, the present study was conducted to provide an in-depth understanding of Iranian adults` perspective towards the concept of positive mental health.

**Materials and Methods**: In the present qualitative study, eight focus group discussions (6 to 8 adults in each session) were held consisting of 30 to 60 year-old men and women from Tehran. Data were analyzed in "DeDoose" qualitative software using content analysis.

**Results**: According to the data obtained, participants found no difference between positive mental health and mental health, mostly equating it to the absence of mental disorders and having positive energy, peace in and satisfaction with life. According to the results, positive mental health has four domains of emotional/psychological, spiritual, social, and life skills.

**Conclusion**: Understanding an individual’s positive mental health concepts culturally and providing appropriate community based programs can significantly promote the mental health of the community.

## Introduction

According to the World Health Organization (WHO), mental health is more than just the absence of mental illness and it is also considered a state of well-being where individuals realize their skills, cope with normal life stresses, can work fruitfully and productively, and are able to make a contribution to their community (see WHO statement on mental health promotion). Based on the results obtained in a study by Keyes *et al.*, complete mental health is a function of two interrelated independent entities, with positive mental health (PMH) as one axis, and the absence of mental disorder as the other ^1– 3^. The complete mental health approach covers previous approaches such as the pathogenic approach, which defines mental health as the absence of mental disorders, and the salutogenic approach, that focuses on the positive aspects of human abilities and functioning in emotions and behaviors ^1^.

Many studies on mental disorders and related diseases have been conducted so far throughout the world. A systematic review study by Steel *et al.*, reported that worldwide one in five adults had experienced a mental disorder during the previous 12 months and 29.2% during their lifetime (lifetime prevalence) in 2014 ^4^. These disorders occur at younger ages, and are chronic, and thus have many adverse effects on various aspects of life, including educational performance, employment, income, personal relationships and social participation ^5^. Thus, there is a need for further, more effective investment into prevention, treatment, and rehabilitation of these disorders.

Despite the advances made in medical and psychiatric therapies, the prevalence and burden of mental diseases has not decreased ^6^. Therefore, community-based interventions should be conducted to prevent the incidence of new cases, and thus promote mental health at a community level ^7^. Using the slogan “mental health for all”, many countries have implemented extensive policies to promote mental health with an emphasis on its positive aspects ^8^. In fact, the concept of PMH includes personal capabilities in psychological, emotional, and social dimensions and promoting PMH leads to a reduction in psychological disorders, a decrease in the probability of all-cause mortality, prevention of suicidal behaviors, and impairment in academic performance ^9– 12^. Many studies suggest that PMH, as a key factor, will lead to positive cognition, positive behavior, and increased cognitive capabilities ^12, 13^.

According to the literature, PMH or mental well-being has two traditional approaches: Hedonic or positive feeling/affects that relies on feeling good (subjective wellbeing, life satisfaction and happiness), and Eudemonic, or positive functioning, with emphasis on optimal social and psychological functioning (engagement, fulfillment, sense of meaning and social wellbeing) ^3, 14^. PMH is formed by the combination of these two approaches, and is conceptualized by the presence of emotional, psychological, and social well-being ^15^. Since this concept is affected by demographic, biological, cultural, socioeconomic and psychological factors etc. ^16, 17^, it occurs very differently in different countries. For instance, the high level of PMH (flourishing) is 11.6% in South Korea, but 76.9% in Canada ^18^. A study conducted by Nosratabadi on Iranian students showed that 16% of students were classified as flourishing ^19^, and a study by Joshanloo *et al.* reported the level of PMH among Iranian students was lower than those in the Netherlands and South Africa ^20^. Understanding and determining the concept of PMH based on cultural-social differences is essential for promoting PMH and preventing psychological disorders. Furthermore, no study has yet addressed this subject in Iranian culture. Therefore, it was decided to conduct a qualitative study to carefully explore the concept of PMH and its domains in the Iranian society.

## Methods

### Study design

The present study was conducted using a qualitative approach, which has proven useful in cultural, psychological, and psychiatric research ^21^. The reason for choosing a qualitative approach was its ability to provide an in-depth understanding of the concept of PMH from the perspective of the society ^21^.

### Participant selection

Data were collected from May to August 2017. Participants were selected by purposive sampling technique ^22^ and with maximum diversity from 30 to 60 years-old Iranian men and women living in different districts of Tehran, capital of Iran. According to purposive sampling and with the aim of obtaining maximum variation of participants` experiences regarding PMH, different characteristics were considered in sampling. These characteristics were gender, age, marital status, level of education, occupation and the social class.

### Data collection

Data was collected using focus group discussions (FGD). This method was chosen for its flexibility, and ability to boost constructive interactions amongst individuals, while increasing the understanding of the concepts being studied ^21^. The number of FGDs was decided according to data saturation, and discussions were performed in single gender groups (men and women separately). Each FGD lasted between 60 and 90 minutes on average, and ended when no new concept could be extracted. Each session was conducted with the participation of 6 to 8 Iranian adults. The locations for group discussions were chosen by interviewees (workplace, tailor shop, municipality health home etc.). In each FGD, first the moderator (corresponding author) introduced herself and her colleague (note taker) and explained the study objectives. A guide questionnaire was used to conduct and direct FGDs. This guide questionnaire had been designed by the research team members using a review of the literature and then validated by group discussion with experts ( Supplementary File 1). After the introduction, FGD began with a general question “What characteristics do you think a person with mental health has? Please explain”. Then, the FGD continued with other questions such as” In your opinion does mental health have any domains?” The concept of PMH and its domains were also asked and debated. During discussions, participants were encouraged to express openly about their opinions and experiences, and probing words such as where, when, how, why, etc. were used to ensure proper understanding of concepts referred to by participants, with the intention of exploring new concepts. The FGD ended when no new topics were expressed.

### Data analysis

All discussions (conducted in Persian) were recorded using a digital voice recorder. Each FGD was transcribed and all field notes and contextual details were added immediately after the session. Data were analyzed using directed content analysis ^23^. By using the Dedoose qualitative software Version 7.6.6, all transcribed discussions were imported into the software as a Microsoft Word template. Dedoose is a powerful, feature-rich, collaborative web-based application for managing, analyzing and presenting qualitative and mixed methods research. The software is applicable for Persian language and illustrates hierarchical linkage of codes for clear visualization of data structure. Discussions were read line-by-line and appropriate codes were assigned by the software. Then, coding was performed according to the study framework and the concept of PMH and its domains. Themes and subthemes extracted were used as the basis for creating new codes or modifying previous ones. Inductive codes were identified according to categories already found. Various aspects of trustworthiness were observed. All results obtained from each group discussion were shared with participants and checked by them (respondent validation). Data analysis was performed by two experts in the research team (dependability). Details of methodology, including collection of data, analysis, coding, etc. were clearly recorded (transferability). Team consistency was observed by the research team members during the process of analysis ^24^.

## Ethics and consent

All participants completed informed consent forms. They were assured that their identities and other related information and responses would remain confidential throughout the survey and after.

Participation was on a voluntary basis, meaning they could leave the FGD at any time during the session. Permission to record the discussions was obtained from participants for appropriate and precise analysis. The study protocol was approved by the ethical committee of the University of Social Welfare and Rehabilitation Sciences (Code: IR.USWR.REC.1396.204).

## Results

In the present study, eight FGDs were conducted with individuals living in Tehran (4 FGDs with men and women separately). A total of 51 adults (29 women and 22 men) took part in these FGDs. The mean age of participants was 46.1. About one fifth of participants were unemployed and 47% had at least one university education. In this study, all individuals who had university education were considered as educated and those with high school certificate and lower degrees were deemed uneducated persons. Demographic variables of FGD participants are shown in Table 1.

**Table 1.** Characteristics of participants in focus group discussions.

Variable	Categories	Female(N=29)	Male(N=22)
Age (year)	Min	30	30
	Max	58	59
	Mean	45.3	46.9
Education (N)	Diploma & lower (uneducated)	18	6
	Higher than Diploma (educated)	11	16
Working status(N)	Employed	14	18
	Housewife	9	0
	Unemployed	6	4
Marital Status(N)	Married	20	14
	Single	5	6
	Divorce	4	2
Number of Children(N)	No child	5	8
	One child	4	4
	Two Children	14	8
	Three & More children	6	2

In our qualitative investigation, two main themes were extracted; namely, general concept of positive mental health and domains of positive mental health, and four subthemes namely a) emotional/psychological domain, b) spiritual domain, c) life skills domain, and d) social domain (see Table 2).

**Table 2.** Subthemes associated with domains of PMH.

Categories	Subcategories
Emotional/Psychological	satisfaction and calmness, feeling of love, empathy, optimism, mutual understanding and respect, personality stability, patience, feeling good, autonomy and flexibility
Spiritual	trust in God, belief in the other world (judgment day), faith in God, connection with God and moral Values
Social	social relations and Interaction, respect other people’s rights, accepting social norms, abiding and respecting law and standing up against injustice
Life skills	anger control, financial management, stress management, accountability, time management, problem-solving

### General concept of PMH

The majority of participants found no difference between PMH and mental health, and in fact considered them absolutely the same. Some believed that PMH meant getting positive energy from family and social environments. In fact, in providing a general definition of PMH, the focus was on positive energies, with both internal and external origins.

*“PMH means absorbing all positive energies…”*(An uneducated woman *). “PMH means having control over your behaviors and solving your problems”*(An educated woman). *“PMH means energizing yourself and enjoying your life …”*(An educated man).

In view of most participants, PMH is a personal attribute that initially begins with being happy and living a happy life, and continues with satisfaction, flexibility, peace, and altruism.

*“I believe it is better to be happy in every situation. Laughing is the treatment of all incurable ailments…”(*An educated man).

Some considered happiness as the logical outcome of being in control of the situation. *“Happiness is brought about by control over different situations. We behave correctly when we are in control”* (An educated man), and women believed: *“PMH rather meant enjoying what you have and being satisfied with your circumstances”*.

The view of many participants was, PMH has social dimensions, as well. Interpersonal relationships, respect for the rights of others, and observing social values are among dimensions that make up PMH.

*“You must behave in such a way to respect the personality of every single person in the community”(*An educated man). *“A mentally healthy person does not do anything that is against the custom”* (An educated woman).

Having different skills was cited to complement the definition of PMH, and it was separately proposed as one of the dimensions of PMH due to its particular importance.

### Domains of PMH

According to participants’ views, we identified four domains of PMH in Iran, namely emotional/psychological, spiritual, social, and life skills in Iranian culture. Overall, these domains consisted of 29 subthemes covering different characteristics of an individual with PMH. Emotional/psychological is the main domain that covers many interlinked attributes of a mentally healthy individual. As an Islamic Asian country, where Iranian culture and Islamic beliefs are intertwined, spirituality and religiousness are considered as an essential element of PMH, which are presented as a separated domain. It also should be noted that developing life skills was mentioned separately due to the importance that participants placed on it ( Table 2).

***Emotional/psychological domain.*** All FGD participants said that emotions and personal characteristics are essential elements of mentally healthy people. They described emotions such as love, kindness, happiness etc., as the software of a human being. One must have suitable and harmonized software and hardware to be defined as a healthy person.

- Satisfaction and calmness: According to participants’ views, satisfaction was one of the most important attributes of those with PMH. Both gender groups believed that satisfaction was a unique attribute that protects an individual against adversities and brings calmness. “ *It is not important what our financial status is; it is important seizing the moment and always feeling satisfied*….”(An educated woman). “ *Financial issues are important; they don’t bring happiness although some people lose their mental stability when they become poor*….” (An uneducated woman).
- Affection was another attribute that was proposed in the emotional domain of PMH. Affection included loving oneself, family, friends, and society leads to kindness and power to support and assist others. “ *To Love yourself, others, family, colleagues, and neighbors without any expectation is very effective. One with PMH should be in love, really in love with others ….* ”(An uneducated man).
- Empathy which was derived from feeling of love cited by participants. Participants believed in it as an inseparable trait of people with good mental health despite of religion or ethnicity “ *An emotionally healthy person cannot rest when he sees someone in trouble; saddens by sadness of others, cheers up with other people’s happiness. Such a person cannot be indifferent, and is affected by other people’s feelings and emotion…*“(An educated woman).
- Expressing feelings and emotions: Mostly women pointed to this characteristic as a coping mechanism encountering unpleasant situations. Men cited to be mentally healthy one should consider logic in his emotion. “ *Anyone who does not consider emotion will lose his/her mental health…*no one likes an apathetic person…” (An uneducated woman).
- Mutual understanding: Most of participants noted it is an essential element in a relationship, especially in family relationships. One with good mental health understands others emotionally and psychologically, and can respect and support them more appropriately.
- Optimism: Many participants agreed that positive thinking and positive feeling creates trust, hope and progress in activities and that is the reason why people interested in optimistic people “Being *positive generates hope, even if it is faked…*” (An educated women).
- Personality stability is another issue in the psychological dimension of PMH. Participants expressed mentally healthy people never change their manner easily and are reliable. In normal situations, everyone has a balanced character while he/she may experience different levels of instability in adversity “ *…if each person has a steady personality in the face with miseries, and maintains his/her balance, that person is a mentally healthy…* ”(Men`s group).
- Feeling good: This characteristic was mentioned in both male and female FGDs “ *feeling good is important, and everyone should find out what makes her/him well. Some feel good by laughing and some by crying. It depends on personal characteristics…*” (An uneducated woman).
- Patience: This dimension also included patience in adversity in all life conditions. A patient person has less expectation and more contentment. *“The threshold of patience and tolerance is very high in a mentally healthy person and he is patient in all adversities* …”(Men`s group).
- Autonomy: “ *Autonomy of personality is very important. Dependent people are very vulnerable*…” (Men`s group).
- Flexibility: Participants believed that flexible individuals experience less stress. “ *Mentally healthy people accept life problems and have a better position to come to terms with them*…” (Women’s group).

***Spiritual domain.*** In people’s view, spirituality is another of PMH’s personal dimension and emerged as a component of it. Spirituality was perceived to be essential in encountering adverse events. Participants mentioned a wide range of virtues based on their religious beliefs and values. They did not differentiate between spirituality and religiousness, although most of them stated their religious beliefs and practices.

- Connection with God: Most women believed that this dimension has a role in the definition of PMH because it strengthens being thankful, contented, and patient, which were recalled as flexibility. “ *When you are connected with God, everything is perfect. People, who are connected with God, can better accept difficult circumstances of life…*” “ *Anyone who seeks help only from God and has a strong connection with God, it means she is perfect….*”
- Faith in God and belief in the other world (Judgment day): Another issue relating to the spiritual domain of PMH is faith which brings forgiveness, feeling of gratitude and peace. “ *Belief in the judgment day and worship are attributes of mentally healthy person*…” (An uneducated woman). *“Being thankful and having faith is the core key. Having faith makes the individual peaceful, and a peaceful person is mentally healthy*….” (An educated man).
- Trust in God: Another issue in this domain is trust in God. Most participants believed that people, who trust in a solid support like God, attain peace and tranquility that guarantees their mental health.
- Moral values: Many moral qualities and values considered attributes of a mentally healthy person were presented by participants in all FGDs like truthfulness, kindness, forgiveness, charity, devotion, thankfulness, composure, helpfulness, etc.

***Social domain.*** All participants believed that individuals with PMH are social people. They enjoy their relationship with others and exchange positive energy. A mentally healthy person can be considered as a supportive resource. Participants expressed different elements of social domain.

- Social relations and interactions: The majority of participants believed this issue should be considered as part of the definition of PMH. Having good interactions and possessing such skills was mentioned in all FGDs. Also, proper relationships were announced as key to providing support. *“Mentally healthy individuals communicate so well that they are said to have ‘the charm’; everybody (young and old) appreciates them, and they can communicate to all age groups. They can always fascinate everybody ….*”(An uneducated woman). “ *A mentally healthy person communicates to others pleasantly… everybody can have his support…*” (An educated man).
- Respect other people’s rights: This characteristic was addressed only by the men’s groups. “ *One should respect other people’s rights, and be able to accept fellow human beings as they are, and not express superiority over others, meaning that one should not be narcissistic…*”
- Acceptance of social norms “ *In my view, a mentally healthy person respects law and lives according to the society’s established norms and customs…*” (Women`s group).
- Abiding and respecting law was another issue proposed in the social dimension, which was cited in the definition of PMH from participants’ perspective.
- Standing up against injustice was another issue proposed in the social dimension of PMH, which was mostly suggested by men. “ *A person is said to have PMH if they stand for their rights in community as much as they can*…”

***Life skills domain.*** In most participants’ views, PMH means having certain life skills which are necessary and help people cope with challenging situations, think positively, and reach their goals in a better way.

- Stress management: The overwhelming majority of participants experienced stress especially in their daily life and considered stress management, or having control over life adversities, as an inseparable part of PMH. “ *In my view, a person is said to be healthy if they can have proper control over life problems or stresses that may occur, whichever side the stress is from…*”( An educated woman).” *In dealing with problems, people should try to find the best solution, but those with the poor mentality cannot accept and overcome the problems and become indifferent…*”( Men`s group).
- Anger control: Another life skill recalled in providing PMH was anger control. In the view of the participants, anyone that can control anger in adversity and react appropriately has sound mental health.
- Financial management: An essential life skill considered by majority participants in order to maintain PMH was to control income and expenses. They believed all people experience different types of financial instable situations. The one who has the power of financial management to control and manage these instabilities in his life more efficiently has good mental health. “… *mentally healthy individuals can manage their personal life in such a way so as to make their family feel fortunate despite financial difficulties…*”( Men’s group)
- Accountability was also cited as an important skill in the definition of PMH. “ *Dutifulness, however defined by the individual is respectable, and a sign of good mental health*…. ” (Women`s group).
- Time management: It was mostly referred to by women as another life skill that affects PMH. *“A mentally healthy person uses her/his time well ….and saves time to help others …*”
- Problem-solving: This skill is among the most important skills pointed out by most participants. *“A healthy person is a kind and sincere person who solves their problems calmly and does not react with haste….* (Men`s group).

## Discussion

The main objective of this present study was to explore PMH definition and its domains in accordance with the Iranian culture. Although this element of mental health seems to be the same in different regions, it is obvious that the concept of PMH is interpreted differently in diverse cultures with respect to its expression and significance ^25^.

The obtained results showed that Iranian participants considered the absence of mental disorder as a prerequisite for mental health and no one considered absence of mental disorder and PMH as two distinguished notions. According to Keyes *et al.*, mental health has two separate but correlating axis – one referring to presence or absence of mental disorders, and the other expressing a high level of PMH to a low level of PMH - and existence of one does not contradict the presence of the other. It means despite having mental disorder, an individual can have some level of PMH or vice versa ^1, 10, 13^. The results of the study implemented by Gilmour in a Canadian community supported Keyes` two continua model ^18^. This theory was not announced by any of the Iranian individuals participating in FGDs. Whereas Laidlaw and his challenges explored in their qualitative study that Scottish people paid attention to PMH and mental health separately ^26^.

In fact, Iranian participants generally considered PMH as getting positive energy, living happily, satisfaction and having peace and flexibility, with four main dimensions of emotional/psychological, spiritual, social, and life skills. Despite the difference in the perception of PMH in different cultures ^14^, our findings appear to comply with the PMH concepts in the literature, especially the WHO description ^5^.

Results of the study revealed four interrelated domains as emotional/psychological, spiritual, social, and life skills in Iranian culture. The findings support Vaingankar`s qualitative research in Singapore, another Asian country with a diverse cultural context, that described PMH domains as personal growth and characteristics, coping strategies, spiritual beliefs and relationships ^25^. Previous studies implemented by Keyes and his colleagues described PMH as salutogenic approaches of subjective wellbeing: with positive feeling defined as emotional wellbeing and positive functioning reflected as psychological wellbeing and social wellbeing ^1, 3^.

The role and importance of satisfaction reasonably creating and maintaining PMH were mentioned in the emotional/psychological dimension. According to the present study results, in the emotional domain, attributes such as satisfaction, love, empathy, understanding feelings and emotions, living happily, and positive thinking were suggested by participants. Review of the literature is also indicative of the importance of satisfaction in the maintenance and promotion of PMH in different cultural countries (see WHO Summary Report on PMH) ^25^. Studies conducted by Monlina and Lyubomirsky indicated the importance of happiness, happy living and other positive emotions in increasing mental/physical well-being ^27, 28^. According to a study conducted in Singapore, positive thinking is able to affect promotion of PMH through interaction with people’s personality attributes ^25^.

In the spiritual domain, virtues such as faith in God and trust in a supernatural force, and also belief in the hereafter, were highlighted by the Iranian participants as with other Asian religious regions ^25, 29, 30^. It seems that spirituality is a notable way of obtaining wellbeing and there is a widespread belief that people with spiritual beliefs and faith have better mental wellbeing ^31, 32^. Although religiosity is considered as a main variable in defining PMH, Ganga and his challenge showed that people with different religions experience diverse level of PMH ^30^. It is important to realize more about interpersonal variations. Previous studies have shown that the most common mechanisms through which religion promotes well-being include social support networks, coping resources and positive emotions ^33, 34^. The spiritual dimension also increases creativity, patience, hope and vitality through communication and coping strategies ^35^.

In the social domain, proper social relations, respect for the rights of others, acceptance of social norms, and lawfulness were cited by Iranian participants. The literature contains evidence on the significant role of proper communication with others and high level of mental well-being ^36^. Close relationships provide support when needed as well as mutual cooperation ^25^. Social support networks have a considerable relationship with mood, performance and the quality of life (see WHO statement on mental health promotion). New theories of well-being also point to the social aspects which correlate with the present study results ^37^.

Regarding life skills, which were explored as one of the main dimensions of PMH; stress management, accountability, anger control, problem solving, financial management, and time management were proposed in the present study. Stress coping skills appear to be one of the most important skills for finding peace and promoting well-being. In fact, stress coping mechanisms are not only important as factors affecting PMH, but also as PMH outcomes ^38^. Studies showed there is a strong relationship between financial management and satisfaction, and mental wellbeing ^39^. Also, existing evidence demonstrates a positive relationship between problem solving skills and social competencies ^40^. Generally, life skills empower people to cope with stresses and emotions, think critically, and are capable of making proper decisions.

The present study has certain strengths and limitations. There is lack of appropriate studies, especially qualitative studies regarding defining social determinants of PMH in Iranian culture which can be considered as one of the most important strengths of this study. Using the qualitative approach provides an in-depth understanding of the concept of PMH from the society perspective. Besides, maximum variation has been observed in the selection of study samples (marital status, occupation, gender and education).

Considering the study limitation, there is lack of generalizability and representativeness since the study was conducted using a qualitative method, and only its process and methodology can be generalized. The findings need more research attempts to be applicable to the broader Iranian adult population due to the multi ethnicity context of the country.

In this study, we interpreted the concept of PMH and its aspects according to Iranian adults’ experiences. The results highlighted the significance and influences of emotional, psychological traits, spiritual and religious beliefs, social aspects and life skills on PMH. Although PMH is considered a well-defined concept, sited as top priority and measured annually in various national mental health surveys in most developed countries, it is a new subject in our nations and needs more investigations and research so it can be introduced as a culturally and linguistically multidimensional concept. Furthermore, a proper understating of how mental health professionals conceptualize the concept of PMH is essential in policy making and implementing proper community-based interventions in order to promote and protect mental health.

## Data availability

The data referenced by this article are under copyright with the following copyright statement: Copyright: © 2018 Mirabzadeh A et al.

Data associated with the article are available under the terms of the Creative Commons Zero "No rights reserved" data waiver (CC0 1.0 Public domain dedication). 

Using Persian language as the national language of Iran during the process of data gathering, all raw data including quotes are available in Persian. Translation and availability of complete raw data will be done upon the request and the permission of Ethical Committee of University of Social Welfare and Rehabilitation Sciences in order to maintain the participants’ confidentiality. Anyone wishing to access the data should first contact the corresponding author who will facilitate contact with the ethical review board ( http://www.uswr.ac.ir Persian site, http://en.uswr.ac.ir/ English site. Contact email international_affairs@uswr.ac.ir)

## Supplementary material

Supplementary File 1: Guide questionnaire in English and Persian.
