# Supplementary material for: Positive Mental Health from the perspective of Iranian society: A qualitative study
Source: F1000Res. 2018 Feb 16;7:103. Originally published 2018 Jan 23. [Version 2] doi: 10.12688/f1000research.13394.2 (PMC5801562; doi:10.12688/f1000research.13394.2)
Supplement: Supplementary file 1 [file f1000research-7-15272-s0000.tgz › da170bdd-0f01-4571-86af-05a04a42181c.zip!/Supplementary File 1/Guide Questionnaire - English.doc]

**Guide questionnaire for Focus Group Discussion**

**Positive mental health from the perspective of Iranian society: A qualitative study**

**General information of the FGD:**

Number of FGD:

Date of FGD:

Participant group:

Place of FGD:

The start time of the FGD

The ending time of the FGD

The number of participants:

**Target Population:** 30 to 60 years-old Iranian men and women living in Tehran

**FGD guide questionnaire**

Introduction and expressing the aim of the FGD

IT is recommended to begin the FGD with a simple and general question

- What characteristics do you think a person with mental health has? Please explain**.**
- In your opinion does mental health have any domains?
- What characteristics do you think a person with positive mental health has? Please explain**.**
- In your opinion does positive mental health have any domains?

Using probes as

What do you mean?

Would please give an example?

Would you please explain more about?

(when, how, why and where etc.)
