# Supplementary material for: Positive Mental Health from the perspective of Iranian society: A qualitative study
Source: F1000Res. 2018 Feb 16;7:103. Originally published 2018 Jan 23. [Version 2] doi: 10.12688/f1000research.13394.2 (PMC5801562; doi:10.12688/f1000research.13394.2)
Supplement: Supplementary file 1 [file f1000research-7-15272-s0000.tgz › da170bdd-0f01-4571-86af-05a04a42181c.zip!/Supplementary File 1/Guide Questionnaire - Persian.docx]

**راهنمای بحث گروهی متمرکز**

**سلامت روان مثبت از منظر جامعه ایرانی**

**اطلاعات کلی بحث گروهی**

شماره بحث گروهی :

تاریخ بحث گروهی :

گروه شرکت کننده:

مکان بحث گروهی :

زمان شروع مصاحبه :

زمان پایان مصاحبه :

تعداد اعضای شرکت کننده در بحث گروهی:

**راهنمای بحث گروهی متمرکز**

معرفی و بیان اهداف مطالعه و بحث گروهی متمرکز

برای شروع مصاحبه پیشنهاد میشود با این سوال کلی و ساده شروع کنید:

- به نظر شما فردی که دارای سلامت روان است دارای چه خصوصیاتی میباشد؟ لطفا توضیح دهید
- به نظر شما سلامت روان دارای چه ابعادی لطفا توضیح دهید؟
- به نظر شما فردی که دارای سلامت روان مثبت دارای چه ویژگی هایی است؟
- به نظر شما آیا میتوان برای سلامت روان مثبت ابعادی در نظر گرفت. لطفا توضیح دهید

به منظور بحث و تبادل نظر و درک عمیق مفاهیم می توانید از سوالاتی مانند " منظور شما از این مطلب چیست"، " لطفا مثال بزنید"، " ممکن است در خصوص مطلبی که عنوان کردید بیشتر توضیح دهید" و همچنین واژه هایی مانند چطور، چه وقت و چرا، استفاده نمایید.
