# Supplementary material for: Positive Mental Health from the perspective of Iranian society: A qualitative study
Source: F1000Res. 2018 Feb 16;7:103. Originally published 2018 Jan 23. [Version 2] doi: 10.12688/f1000research.13394.2 (PMC5801562; doi:10.12688/f1000research.13394.2)
Supplement: Peer review history

# Peer review: Referee response for version 1

**DOI:** 10.5256/f1000research.14542.r30133

**Published:** 2018-02-05

**Authors:** Mitra Modirian

**Affiliations:** 1 Non–communicable Diseases Research Center, Endocrinology, Metabolism Population Sciences Institute, Tehran University of Medical Sciences, Tehran, Iran

Present paper revealed a well-designed study entitled “Positive Mental Health from the perspective of Iranian society: A qualitative study” through which benefitting from a qualitative design, data gathered by focus group discussions.

 Requirements for qualitative studies have been followed completely and results and extracted conclusion presented in practical format.

 Some notes:

Sampling should be explained more with details of referred technique.

Discussion: the participants were residents of capital city of Iran, so the society of participants was different from other Iranians which should be notified. Also the negative points and limitation of study should be clearly more explained, there are more social and individual factors (social category and class, financial support, family of each participants, awareness of quality and quantity of insurance-services for which may be focused due to interruption with results of present study, but there was no notification of them in discussion or conclusion.

 These findings also could be used for future complementary researches.

I have read this submission. I believe that I have an appropriate level of expertise to confirm that it is of an acceptable scientific standard.

# Peer review: Referee response for version 1

**DOI:** 10.5256/f1000research.14542.r30134

**Published:** 2018-02-05

**Authors:** Maryam Tehranizadeh

**Affiliations:** 1 Payame Noor University, Karaj, Iran

Thanks for sharing this manuscript with me. Present study focus on positive mental health from the perspective of Iranian society. It is a qualitative investigation, conducted through focus group discussions.

 The methods section write carefully and extracted results present in appropriate format. Only as minor comments please follow bellow points:

1. Referring to related reference, present more details on Dedoose qualitative software Version 7.6.6 applications
2. Add more detail about development the guide questionnaire
3. Add the main *strengths* *and limitation to the end part of discussion*

I have read this submission. I believe that I have an appropriate level of expertise to confirm that it is of an acceptable scientific standard.
